# Society Meetings

**Published:** 1915-11

**Authors:** 


### SOCIETY MEETINGS

---

**Brief reports and announcements of meetings of societies of Western New York, and those of general scope, are requested from Secretaries. Copy should be on hand the fifteenth of the month. Full transactions will be published at cost of composition.**

---

**DOCTOR: Is your Society properly represented here? If not, please bring our standing announcement to the attention of the members.**

---

At the monthly meeting of the **Medical Society** of McKean County, Pennsylvania, in November, by invitation Dr. J. Henry Dowd will read a paper on, "The Care and Treatment of Our Neurotics."

---

**The Gross Medical Club** held their stated meeting at Tonawanda, Friday, Oct. 15th, being entertained by Dr. R. H. Wilcox.

A most interesting and instructive paper, "Some phases of Public Health work," was presented by Dr. W. H. Baker, of

Williamsville. Dr. Baker, who is a very forcible speaker, brought out most clearly what is expected of a Health Commissioner in the rural districts, and what can be done by him, providing he has the moral courage to insist, laws and rules he knows he has the authority to enforce, are so enforced.

This paper was most thoroughly discussed by several of the members, all health officers of surrounding rural localities. The consensus of opinion was; Health affairs should be entirely divorced from politics, in this way, much could be gained and favoritism could be entirely banished.

Several important cases were reported:

Dr. Lawrence Smith, East Aurora, a boy of five or six whose mother said "He jerked (convulsive evidently) in his sleep." He passed only three or four ounces of urine in twenty-four hours; which contained casts and albumin. Under appropriate treatment he apparently recovered. In about two or three months, he was suddenly seized with a twitching of one leg and great weakness. Examination of the blood showed typhoid, yet he had no symptoms or temperature: finally paralysis involved the left leg. In consultation later, the case was diagnosed as Tubercular Meningitis. Suffice to say, after several weeks of a stationary condition of symptoms the boy is now recovering quite rapidly.

Dr. Chester C. Cott. The case of a sailor with severe pains in ear, which in a few days was followed by chills. An operation revealed pus in the right mastoid cells. Two or three days later, chills reappeared and the lateral sinus was open, but no pus found here. Chills continued and he died. A post-mortem revealed an extra dural abscess under the petrous portion of the temporal bone.

Dr. B. E. Smith. A man aged 58, urinating twelve times at night and every half hour in the day. Prostate normal, per-rectum, no stricture. His walk was characteristic of marked locomotor ataxia, but petellas reflexes were exaggerated. His conversation and every action pointed to dimensia: he had been ailing for six or eight weeks, being treated with no result. Urinary examination, sp. g. 1022, albumin, pus, but no other pathological findings: Phosphatic Index 175% plus. The condition appeared most deplorable, there was a grave question as to the outcome. Viewing it as a high state of nerve-cell irritation, as shown by the Dowd Phosphatometer, Fl. Ex. Valerian in Uriseptin was ordered three times a day. I did not see him again for fourteen months, and imagine my surprise to be told, all symptoms had disappeared in three days, he had gone back to work, and has been feeling perfectly well since. I report this case simply to show how, when the true cause is shown, how easily and quickly an apparent serious condition can be handled.

Dr. Wurtz, Kenmore. A woman, extreme lassitude, general pains throughout the body, morning temperature 97, P. M. 100, this for ten days, constipated. There was a very foetid odor from the skin, negative typhoid and Wasserman. For three days past, temperature normal, but she cannot get up or practically move on account of the pain in the muscles, this case is not rheumatism.

Dr. J. Henry Dowd. Patient with pimples under arm, mole-like in appearance, bled easily if touched. This was removed by Dr. James E. King, who also removed all the glands in the adjacent region. It proved to be a melanotic sarcoma. Six months afterwards, complained of pain in the neck with swelling, also under the ribs, the side being markedly bulged. The swelling in the neck is a sarcoma and no doubt the one under the ribs is the same. This is evidently a case of general sarcomatosis.

The members were given quite a treat in a brief talk by Mr. Edward Britt, brother of Dr. W. W. Britt, an expert aviator with the Curtiss Company.

---

**The Association of Erie Railroad Surgeons** held its 24th annual meeting in Buffalo, Sept. 30 and Oct. 1, with an attendance of about 100. Papers were read and a clinic was given by Dr. Wm. H. Mansperger at the German Deaconess Hospital. The Erie R. R. entertained the members at a banquet at the Iroquois, followed by a theatre party and with a trip to Niagara Falls. The President, Dr. J. D. Zwetch of Gowanda, presided.

---

**The Medical Society of the County of Erie** held its regular meeting in Townsend Hall, Niagara Square, Buffalo, Oct. 18. Officers for 1916 were nominated as follows: Pres., Franklin W. Barrows; 1st V. P., Irving W. Potter; 2d V. P., John C. Thompson; Sec., Franklin C. Gram; Treas., A. T. Lytle; Censors, John D. Bonnar, Arthur G. Bennett, A. D. Carpenter, Frank A. Valente; Delegates to State Society, Arthur G. Bennett, Francis E. Fronczak, A. T. Lytle, Julius Richter, Franklin W. Barrows; Chairmen of Committees, Legislation, Harvey R. Gaylord; Public Health, John H. Pryor; Membership, Wm. F. Jacobs; Economics, John V. Woodruff.

The following new members were elected: Anthony J. Hey, Richard M. De Niord, John George Grotz, George G. Cook, Francis M. Welch, O. J. Oberkarcher, Ernest G. Kramer, Henry D. Duryea, John C. Grabau.

Papers were read by Dr. Franklin W. Barrows on the Study of Backward and Delinquent Children and by Dr. Franklin C. Gram on What Shall Be Done with Tuberculous Patients after their Return from Hospitals and Sanatoria.

**Buffalo Academy of Medicine.** Oct. 6, Surgical Section, Dislocation of the Semilunar Bone, E. L. Bebee, with demonstration of case. Dr. C. E. Congdon reported a case of Dislocation of Trapezoid. Color Photography in Medicine and Art, with description of methods and lantern slides, Dr. C. W. Bethune.

Oct. 13, Medical Section, Review of Scientific Progress in Physiology, Dr. F. H. Pratt; Psychoanalysis, Dr. G. A. Sloan.

Oct. 20, Section of Obstetrics and Gynaecology, Medicine in Japan, Dr. Rudolph D. Teusler, St. Luke's Hospital, Tokyo.

---

**Rochester Medical Association,** Oct. 6, Diagnostic Problems, Treatment of Gastric and Duodenal Ulcers, Dr. Thomas Jameson; Early Diagnosis of Gastric Cancer, Dr. John R. Williams.

---

**RochesterMedicalAssociation,** Oct. 6, Diagnostic Problems, Dr. George F. Laidlaw of N. Y.

---

**Rochester Pathological Society,** Oct. 7, President's Address, Dr. Charles L. Hinchner.

---

**The Medical Association of Central N. Y.** held its 47th annual meeting in the Home of the Rochester Medical Association, 33 Chestnut Street, October 21, under the presidency of Dr. C. O. Boswell of Rochester. The program was as follows, Dr. Elsner being prevented by sickness from attending or preparing his paper, Dr. Colton being absent but his paper being read by the Secretary:

President's Address, Charles O. Boswell, Rochester; Therapeutic Uses. Human Blood Serum (by invitation) Joseph Roby, Rochester; Place of Bone Graft in Surgery (with lantern slides) Howard L. Prince, Rochester; Ductless Glands and a Typical Growth (by invitation) Seelye W. Little, Rochester; Business meeting; Luncheon, 1 p. m.

Symposium on Cardio-Vascular Disease: (1) Modern Methods of Investigation of Cardio-Vascular Diseases, Drs. Geo. W. Ross and Julian London, Toronto, Ont., (by invitation); (a) The Polygraph (with lantern slides); (b) Blood Cultures; (c) The Wasserman Reaction; (d) A Method of Auscultation of the Pupular Veins, Dr. Ross; (e) The Electro Cardiograph (with lantern slides), X-Rays (with lantern slides) Dr. London.

Roentgen Ray Diagnosis of the Heart and Lungs with special reference to new growths (with lantern slides) M. B. Palmer, Rochester; Cardio-Vascular Changes with the Nephropathies, H. L. Elsner, Syracuse; Treatment of Cardio-Vascular-Renal Disease, Charles G. Stockton, Buffalo; Tuberculosis as We Should View It, A. J. Colton, Buffalo.

The following officers were elected, the Secretary, Dr. J. J.

Buettner of Syracuse holding over: President, A. L. Benedict, Buffalo; 1st V.-P., Ledra Heazlett, Auburn; 2d V.-P., E. J. Wynkoop, Syracuse; Treasurer (re-elected), T. F. Laurie, Auburn. The next meeting will be held in Buffalo in October, 1916.

---

**The Orleans County Medical Society** held its annual meeting in Medina October 5. Officers were elected as follows: President, John Dugan, Albion; V.-P., R. E. Brodie, Albion; Sec. and Treas., Geo. Rogan, Medina; Censors, Drs. Munson, Padelford and Ogden; Delegate to State Society, John Dugan. Dr. Frank Brundage of Buffalo presented a paper on Rickets in Breast Fed Infants, and Dr. Clayton M. Browne of Buffalo on Preventable Deafness.

